# Extragastrointestinal stromal tumors: Computed tomography and magnetic resonance imaging findings

**DOI:** 10.3892/ol.2014.2705

**Published:** 2014-11-12

**Authors:** JINGQI ZHU, ZHANGWEI YANG, GUANGYU TANG, ZHONGQIU WANG

**Affiliations:** 1 Department of Radiology, Shanghai Tenth People’s Hospital, Tongji University School of Medicine, Shanghai 200072, P.R. China; 2 Department of Radiology, East Hospital, Tongji University School of Medicine, Shanghai 200120, P.R. China; 3 Department of Radiology, Affiliated Hospital of Nanjing University of Traditional Chinese Medicine, Nanjing, Jiangsu 210029, P.R. China

**Keywords:** extragastrointestinal stromal tumors, computed tomography, magnetic resonance imaging, mitotic count

## Abstract

Extragastrointestinal stromal tumors (EGISTs) are rare mesenchymal tumors that originate outside the gastrointestinal tract. The aim of the present study was to investigate the computed tomography (CT) and magnetic resonance imaging (MRI) features of EGISTs and analyze the correlations between radiological findings and pathological features. CT and MRI images of 24 patients with EGISTs were reviewed retrospectively. Patient demographics and tumor characteristics, including localization, size, contours, borders, cystic-necrotic components, calcification, hemorrhage, tumor vessels, attenuation and intensity, degree and pattern of enhancement, ascites, tumor invasion, lymphadenopathy and distant metastasis were recorded. Statistical analyses to compare the radiological characteristics of low- and high-grade EGISTs were performed with χ^2^ or Fisher’s exact tests. The mean patient age at the time of presentation was 53 years. A total of 24 EGISTs were detected, originating in the omentum (n=4), mesentery (n=19) and retroperitoneum (n=1), respectively. The EGISTs displayed a predominantly spindle cell subtype (87.5%; 21/24). The majority of the EGISTs appeared as large (>10 cm; 70.8%; 17/24), round or oval (66.7%; 16/24), cystic-solid (87.5%; 21/24) and ill-defined (66.7%; 16/24) soft-tissue masses. The EGISTs were hypodense (69.6%; 16/23) or isodense (30.4%; 7/23) on CT images, hypointense (50%; 3/6), isointense (33.3%; 2/6) or hyperintense (16.7%; 1/6) on T1-weighted imaging (T1WI), and hyperintense on T2WI (100%; 6/6) and diffusion-WI (DWI; 100%; 6/6). A total of 54.2% (13/24) of the EGISTs displayed tumor vessels. Overall, 95.8% (23/24) of the masses showed marked enhancement and 87.5% (21/24) demonstrated heterogeneous enhancement. Calcification, hemorrhage, ascites and lymphadenopathy were rare characteristics in the EGISTs. Distant metastases were present in 10 patients (41.7%). The size, borders, tumor vessels and distant metastasis correlated with high-grade EGISTs [>5 mitoses/50 high-power fields (HPFs)] (P<0.05). The results of the present study indicated that clinical and radiological features, including advanced age, a large tumor size, cystic-necrotic components, rare lymphadenopathy, a heterogeneous enhancement pattern and hepatic metastasis may aid in the diagnosis of EGISTs. Radiological characteristics, such as a large size (>10 cm), ill-defined borders, tumor vessels and distant metastasis, can provide useful information in identifying the malignant behavior of EGISTs.

## Introduction

Gastrointestinal stromal tumors (GISTs) are the most prevalent mesenchymal neoplasms of the GI tract. The annual incidence of GISTs is reported to be 7–19 cases per million individuals (1). Typically, GISTs arise from the muscularis propria in the wall of the GI tract, and are believed to originate from the interstitial cells of Cajal, the majority of which are positive for KIT [cluster of differentiation (CD)117] and tend to be positive for CD34. The current histological classification for GISTs includes spindle, epithelioid and mixed cell subtypes (2). The stomach is the most common location for GISTs to occur (60–70%), followed by the small intestine (20–30%), colorectum (10%) and esophagus (<5%) (3). However, a small number of mesenchymal tumors with similar histopathological and immunohistochemical characteristics to GISTs have been increasingly described in the omentum, mesentery and retroperitoneum (<7%). These are known as extragastrointestinal stromal tumors (EGISTs) (4–6).

The computed tomography (CT) and magnetic resonance imaging (MRI) features of GISTs have been reported previously. Numerous studies have identified malignant imaging signs for GISTs, such as a large size, irregular surface, ill-defined margins, tissue invasion, distant metastasis, peritoneal dissemination and satellite nodules (7–10). However, few studies describe the radiological findings of EGISTs (3). The purpose of the present study was to review the CT and MRI images of EGISTs and analyze the correlations between the radiological findings and pathological features.

## Patients and methods

### Subjects

The clinical, pathological and radiological findings of 24 patients with primary EGISTs who were treated at East Hospital, Tongji University School of Medicine (Shanghai, China) and Shanghai Tenth People’s Hospital, Tongji University School of Medicine (Shanghai, China) between May 2004 and August 2013 were reviewed. All EGISTs were histologically proven by surgery. The criteria for diagnosing the EGISTs were as follows: i) The mass had no definite connection with the GI tract wall by intraoperative or pathological observations; ii) the mass had typical GIST morphology, as observed by light microscopy; and iii) the mass expressed KIT and/or CD34. Written informed consent was obtained from all patients.

### CT and MRI technique

In total, 23 of the 24 patients underwent CT scans of the abdomen and/or pelvis at the time of presentation. CT examinations were performed with a 16-slice spiral CT scanner (Sensation; Siemens Medical Solutions, Erlangen, Germany; n=12) or a 64-sclice spiral CT scanner (Philips Brilliance; Philips Medical Systems, Best, the Netherlands; n=11). The main parameters of the CT scans were as follows: Tube voltage, 120 kVp; tube current, 250 mAs; slice thickness, 3–8 mm; field of view, 350 mm; matrix, 512×512; gantry speed, 0.75 sec/rotation; and pitch, 1.0–1.2. Oral diatrizoate meglumine (concentration, 3%; dose, 800–1,000 ml; Gastrografin; Bayer Schering, Berlin, Germany) was administered to ten patients prior to the scans. Dual-phase dynamic contrast enhancement was performed to obtain images of the arterial phase (30–35 sec) and venous phase (65–70 sec) subsequent to the intravenous administration of contrast agent (Omnipaque 300; Nycomed Amersham, Princeton, NJ, USA; dose, 1.5 ml/kg body weight; injection rate, 2.5–3.5 ml/sec). Multiplanar reformation and maximum intensity projection images were achieved at an affiliated workstation.

Six out of 24 patients underwent abdominal and/or pelvic examinations with a 3.0-Tesla MRI scanner (Philips Achieva; Philips Medical Systems) using a body coil. The main parameters of the MRI examination were as follows: Field of view, 375 mm; matrix size, 252×192; and slice thickness, 3–6 mm. T1WI [spin echo sequence; repetition time (TR)/echo time (TE), 500/7.9 msec; number of signal averages (NSA), 2], T2WI (fast spin echo sequence; TR/TE, 3,000/65 msec; NSA, 2) and DWI (EPI sequence; TR/TE, 1,147/70 msec; NSA, 2; b value, 800 sec/mm^2^) were obtained in the axial plane, and T2-weighted short time inversion recovery images (TR/TE, 1,822/60 msec; NSA, 2) were obtained in the axial, coronal and sagittal planes. Following the intravenous administration of gadopentetate dimeglumine (Magnevist^®^; Bayer Schering, Berlin, Germany; dose, 0.1 mmol/kg body weight; injection rate, 1.5 ml/sec), dual-phase dynamic contrast enhancement was performed to obtain fat-saturated T1WI (fast field echo sequence; flip angle, 10°; TR/TE, 4.1/2.0 msec; NSA, 2) of the arterial phase (30 sec) and venous phase (60 sec) in the axial, coronal and sagittal planes. In five out of 24 patients, both CT and MRI images were available.

### Imaging and pathological analyses

On the CT images, the attenuation of each tumor was recorded as a hypo-, iso- or hyperdensity compared with the adjacent muscle. On MRI images, the signal intensity of each tumor was recorded as a hypo-, iso- or hyperintensity compared with the adjacent muscle. The cystic-necrotic component was defined as the center of the tumor having a density of <20 Hounsfield units (HU) on contrast-enhanced images or water-like signal without enhancement on MRI images. The radiological images were used to measure the largest dimension of each tumor. The degree of tumor enhancement was classified as mild (<30 HU) or marked (≥30 HU). The enhancement patterns were recorded as homogeneous or heterogeneous. Tumor vessels were defined as engorged vascular structures within the mass. Lymphadenopathy was determined as present if a nodular soft-tissue lesion existed that was >10 mm in the short-axis diameter.

Two experienced radiologists who were blinded to the pathological results of the EGISTs retrospectively reviewed the radiological images, and the findings were reported as a consensus of opinion. Tumor characteristics, including localization, size, contours, borders, cystic-necrotic components, calcification, hemorrhage and tumor vessels, were recorded. The attenuation and intensity, as well as the degree and pattern of enhancement of the EGISTs were evaluated. Radiological findings were also evaluated for ascites, tumor invasion, lymphadenopathy and distant metastasis.

The pathological findings in the surgical specimens were retrospectively reviewed by one experienced pathologist, with a particular emphasis on the presence of morphology, mitotic activity and the immunoreactivity of KIT and CD34. On light microscopy, ≤5 mitoses/50 high-power fields (HPFs) is generally considered to indicate a low-grade EGIST, whereas >5 mitoses per 50 HPFs is generally considered to indicate a high-grade EGIST (1,7). This was also the grading system used for the present study.

### Statistical analysis

Quantitative variables are expressed as the mean ± standard deviation (SD) and categorical variables are expressed as frequencies or percentages. Statistical analyses to compare the radiological characteristics of EGISTs of differing grades were performed with χ^2^ or Fisher’s exact tests (SPSS, version 13.0; SPSS, Inc., Chicago, IL, USA). P<0.05 was considered to indicate a statistically significant difference.

## Results

### Clinical and pathological features

Based on the diagnostic criteria, 24 surgically resected EGISTs were identified. A slight male predominance (13 males and 11 females) existed within the study group. The mean age at the time of presentation was 53 years (SD, 13 years; range, 34–81 years). The mean tumor size was 12.8 cm (SD, 5.3 cm; range, 4.5–25.1 cm). The clinical symptoms were an abdominal or pelvic mass (n=14); abdominal pain (n=8) and abdominal distension (n=7). The primary EGISTs occurred in the omentum (n=4; 16.7%), mesentery (n=19; 79.2%) and retroperitoneum (n=1; 4.2%). The pathological subtype of the 24 EGISTs was classified as spindle cell (n=21; 87.5%), epithelioid cell (n=1; 4.2%) and mixed cell (n=2; 8.3%). Immunohistochemistry showed that 91.7% (22/24) and 70.8% (17/24) of the tumors were positive for KIT and CD34, respectively. Two KIT-negative EGISTs were both of mesenteric origin and epithelioid or mixed cell subtype. The clinical data, pathological subtypes and immunohistochemical results are shown in Tables I and II. According to the mitotic counts, seven (29.2%) EGISTs were of low grade and 17 (70.8%) were of high grade.

### CT and MRI findings

**Figure 1:**
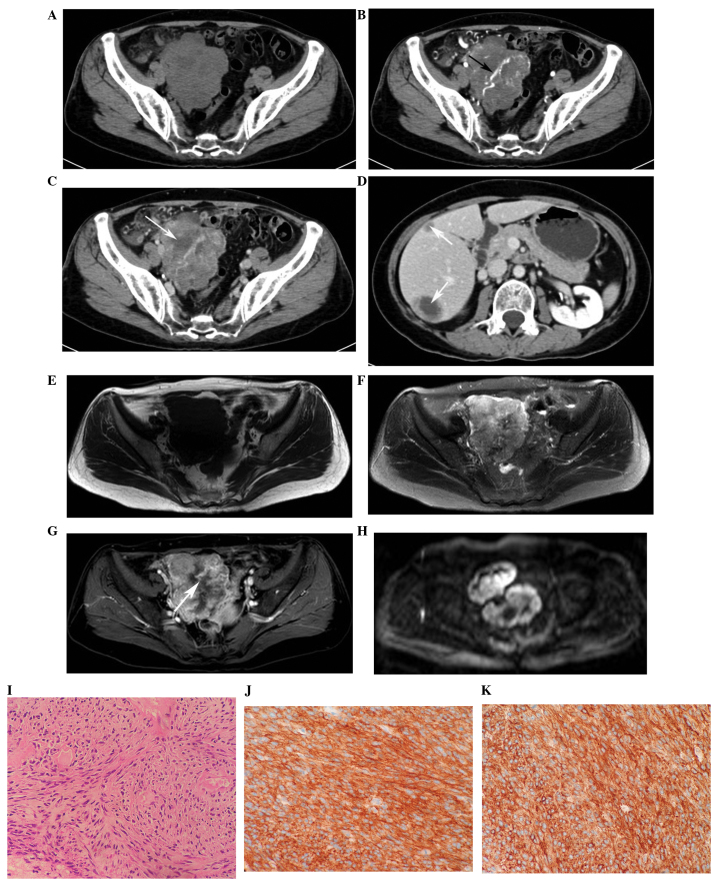
A 56-year-old female with high-grade extragastrointestinal stromal tumor in the omentum. (A) Axial plain computed tomography (CT) image showing an 11.1-cm slightly hypodense mass with irregular contours. (B) Axial CT image showing engorged tumor vessels (arrow) within the mass (arterial phase). (C) Axial CT image showing heterogeneous enhancement (arrow) of the mass (venous phase). (D) Axial CT image showing multiple hepatic metastases (arrows) with peripheral enhancement (venous phase). (E) Axial T1-weighted imaging (WI) showing the mass with slight hypointensity. (F) Axial T2WI showing the mass with heterogeneous hyperintensity. (G) Transverse contrast-enhanced T1WI showing markedly heterogeneous enhancement of the mass with a cystic-necrotic component (arrow). (H) Axial DWI showing the mass with hyperintensity. (I) Photomicrograph of histological specimen showing that a tumor consisting mostly of spindle cells with a high mitotic count (7 mitoses/50 high-power fields; hematoxylin and eosin staining; magnification, ×200). (J) Immunostaining demonstrating positivity for KIT (magnification, ×200). (K) Immunostaining demonstrating positivity for cluster of differentiation 34 (magnification, ×200).

**Figure 2:**
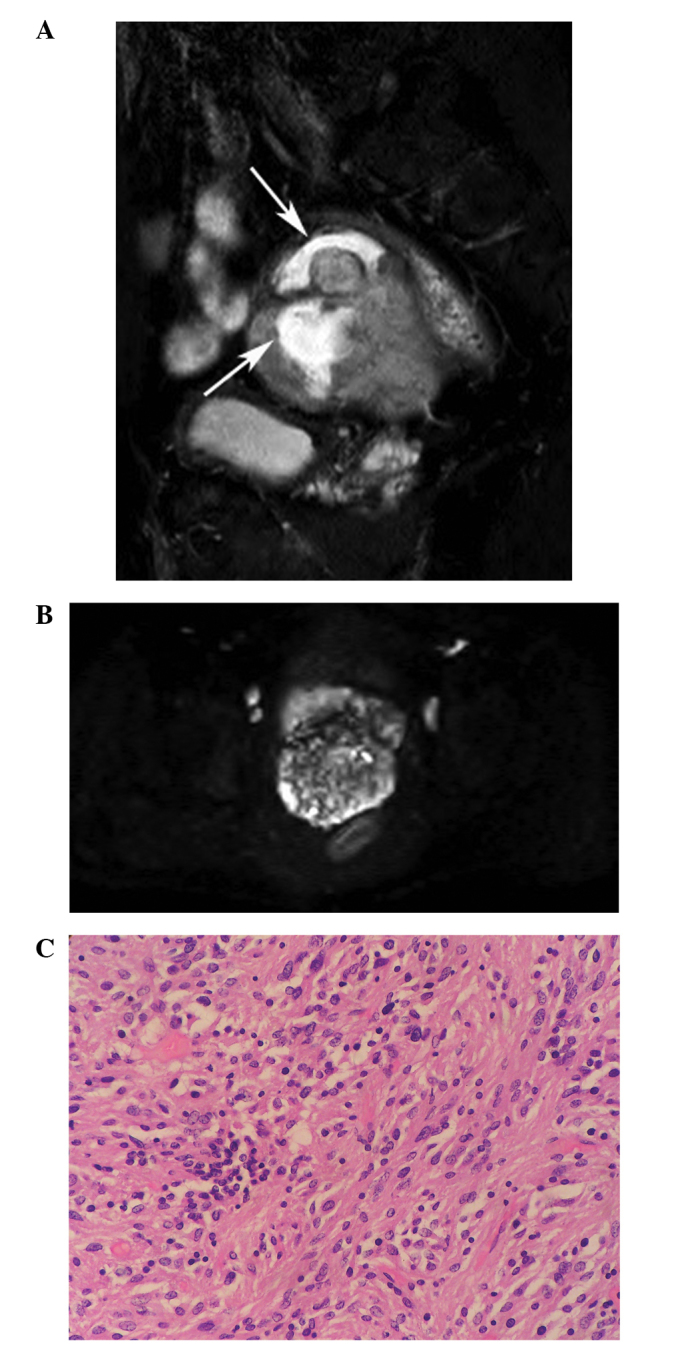
A 60-year-old male with a low-grade extragastrointestinal stromal tumor in the mesentery. (A) Sagittal T2-weighted imaging (WI) showing a 12.4-cm oval mass in the pelvic cavity. Portions with marked hyperintensity (arrows) are apparent inside of the mass, representing a cystic-necrotic component. (B) Axial diffusion-WI showing the mass with hyperintensity. (C) Photomicrograph of a histological specimen showing that the tumor cells are composed of spindle cells with a low mitotic count (2 mitoses/50 high-power fields; hematoxylin and eosin staining; magnification, ×200).

**Figure 3:**
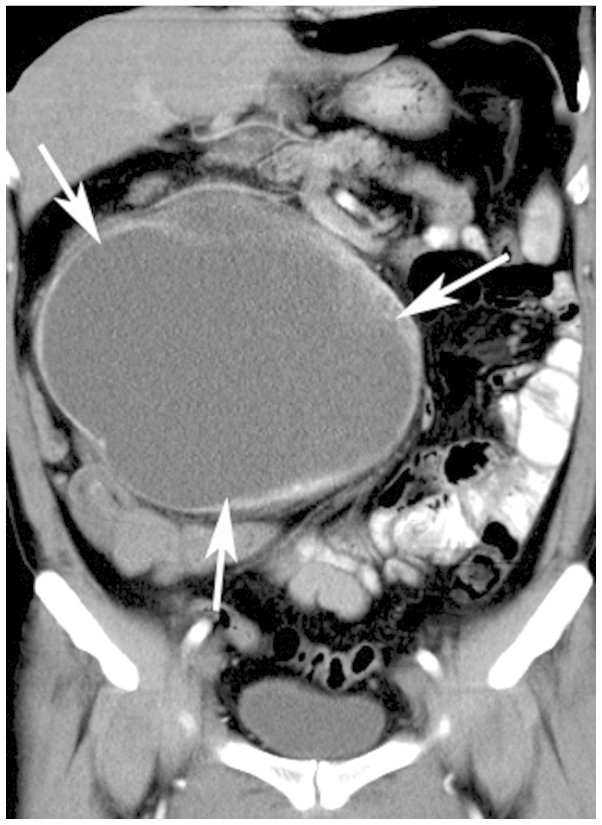
A 46-year-old male with a high-grade extragastrointestinal stromal tumor in the mesentery. Coronal contrast-enhanced computed tomography image showing a 19.3-cm cystic mass with markedly peripheral enhancement.

**Figure 4:**
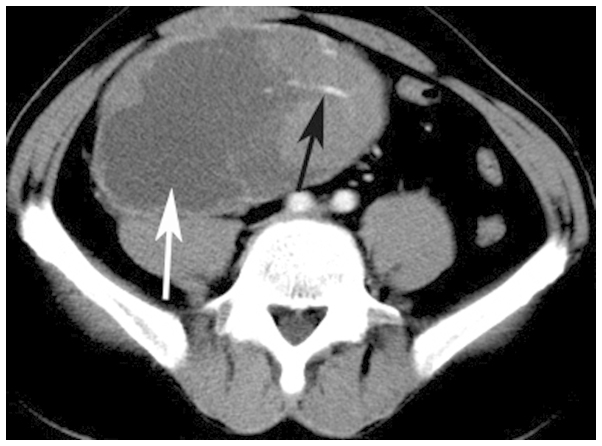
A 50-year-old male with a high-grade extragastrointestinal stromal tumor in the mesentery. Axial contrast-enhanced computed tomgraphy image showing a mass with cystic area (white arrow) and engorged vascular structures (black arrow).

**Figure 5:**
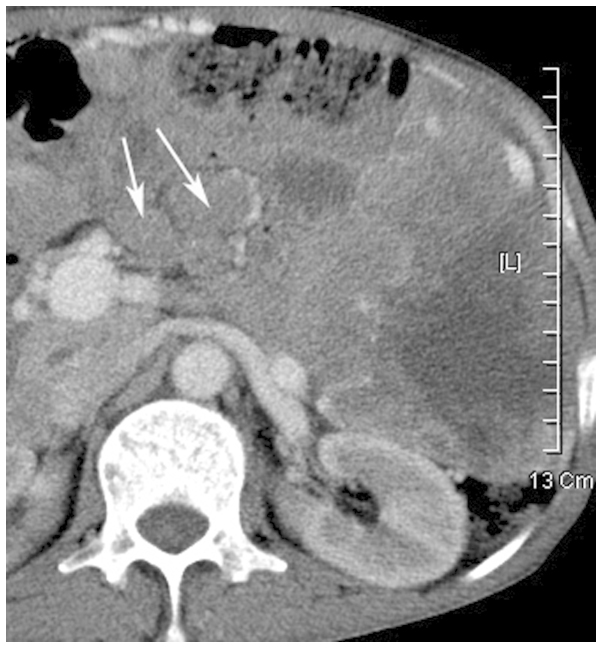
A 64-year-old male with a high-grade extragastrointestinal stromal tumor in the mesentery. Axial contrast-enhanced computed tomography image showing a 25.1-cm, irregular and ill-defind mass with heterogeneous enhancement. Enlarged mesenteric lymph nodes are also observed (arrows).

**Figure 6:**
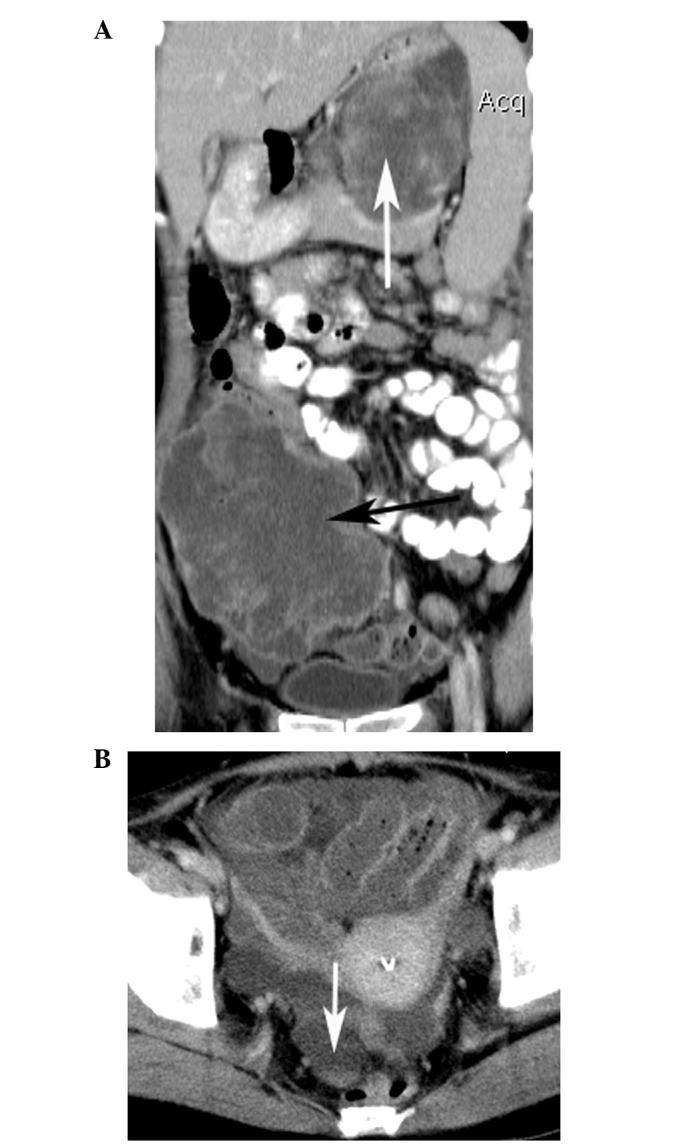
A 42-year-old female with a high-grade extragastrointestinal stromal tumor in the mesentery. (A) Coronal contrast-enhanced computed tomography (CT) image showing a primary mass in the ileocecal region (black arrow) and a metastasis in the adrenal gland (white arrow) with heterogeneous enhancement. (B) Axial CT image showing pelvic effusion (arrow).

**Figure 7:**
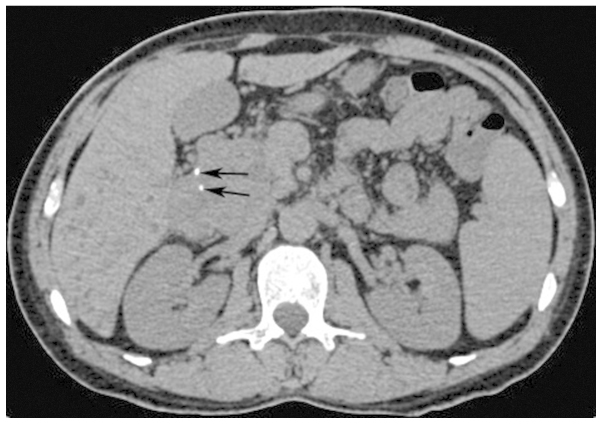
A 46-year-old male with a low-grade extragastrointestinal stromal tumor in the mesentery. Axial plain computed tomography image showing a 5.2-cm isodense mass with calcification (arrows).

On the CT (n=23; seven low-grade EGISTs and 16 high-grade EGISTs) and MRI (n=6; three low-grade EGISTs and three high-grade EGISTs) images, 16 tumors (66.7%) exhibited round or oval contours and eight (33.3%) showed an irregular appearance. The masses were regarded as ill-defined in 16 patients (66.7%). The tumors appeared as a hypodensity (n=7), slight hypodensity (n=9) or isodensity (n=7) on precontrast CT images, as a slight hypointensity (n=3), isointensity (n=2) or slight hyperintensity (n=1) on T1WI, and as a hyperintensity (n=6) on T2WI and DWI (Figs. 1 and 2). A total of 21 tumors (87.5%) showed a cystic-necrotic component (Figs. 1–5). Only one tumor showed mild enhancement, while the others (n=23; 95.8%) demonstrated marked enhancement (Figs. 1 and 3–6). Overall, 21 tumors (87.5%) showed heterogeneous enhancement in the arterial and venous phases (Figs. 1 and 4–6). Calcification was found in one tumor and hemorrhage in two tumors (Fig. 7). Engorged tumor vessels were apparent in 13 masses (54.2%; Figs. 1 and 4). Ascites was observed in three patients (12.5%; Fig. 6B). Enlarged mesenteric lymph nodes without necrosis were observed in one tumor and were proved to be metastases during surgery (Fig. 5). Distant metastases were present in 10 patients (41.7%). The locations of metastases were the adrenal gland alone (n=1; Fig. 6A), the liver alone (n=8; Fig. 1D), and the liver and peritoneum (n=1). All metastases were heterogeneously enhanced and the hepatic metastases were peripherally enhanced with necrotic centers (Figs. 1D and 6A).

### Correlations between tumor grade and radiological findings

Statistical analyses showed that tumor size (P=0.041), tumor borders (P=0.021), tumor vessels (P=0.023) and distant metastasis (P=0.019) correlated with high-grade EGISTs. However, tumor localization, tumor contours, cystic-necrotic components, calcification, hemorrhage, degree and pattern of enhancement, ascites and lymphadenopathy did not exhibit significant differences (P>0.05) between the low- and high-grade EGISTs. The radiological findings of the EGISTs of differing grades are summarized in Table III.

## Discussion

The interstitial cells of Cajal, pace-maker cells that control GI track peristalsis and express the KIT antigen, are believed to be the origin of GISTs. The occurrence of GISTs as primary tumors in extragastrointestinal intra-abdominal tissues, such as the mesentery, omentum, retroperitoneum, abdominal wall, gallbladder, pancreas and rectovaginal septum, occurs rarely (3,4,11–13). Due to the similar histological appearance and immunophenotype compared with GISTs, EGISTs are believed to be representations of either GISTs that have separated from the GI tract wall or independent mesenchymal cell growth of the mesentery, omentum and retroperitoneum (5,14). The incidence of EGISTs is uncertain with regard to gender (3,4,13,15–17). The present study exhibits a slight male predominance (54.2%; 13/24). However, EGISTs occur predominantly in adults, with a mean age of between 50 and 60 years (4,13,16). In the present study, all patients were of an advanced age (mean age, 53 years old), with none being children or adolescents. Previous studies have shown that the majority of EGISTs are large when first diagnosed, with a mean size ranging between 10 and 18 cm (4,13,16). Small EGISTs rarely produce symptoms due to their atypical site. EGISTs are often diagnosed incidentally during investigations for other symptoms. In the present patient group, 70.8% (17/24) of the EGISTs were >10 cm and the most common clinical symptoms, including an abdominal or pelvic mass, abdominal pain and abdominal distension, were non-specific. EGISTs have a predilection for the areas of the mesentery (22.2–42.9%), omentum (25–28.6%) and retroperitoneum (10.7–33.3%) (4,13,16). In the present study, EGISTs in the mesentery, omentum and retroperitoneum were involved in 79.2, 16.7 and 4.2% of cases respectively.

The histopathological appearance of EGISTs is variable, but, in general, three subtypes, including spindle, epithelioid and mixed cell types, are noted. In the present study, the EGISTs predominantly displayed the spindle cell subtype (87.5%; 21/24), which is consistent with previous studies (13,16,17). KIT is overexpressed at a high frequency (96.4–100%) when detected by an immunohistochemical method and has been shown to be a good immunomarker for diagnosing EGISTs (16,18). Thus, KIT-negative EGISTs are rare and their clinicopathological features have not been well documented (2,16,19). Yamamoto *et al* (2) reported that a preference for an omental origin and an epithelioid cell subtype characterized KIT-negative EGISTs. The present study showed that 91.7% (22/24) of the tumors were positive for KIT, with only two KIT-negative EGISTs, both of mesenteric origin and of epithelioid or mixed cell subtype. CD34 staining was positive in 70.8% of the tumors, which is similar to the values previously reported in EGISTs (4,15). The accurate risk stratification of EGISTs has become increasingly important owing to emerging adjuvant imatinib therapy. Based on GIST size and mitotic count, the National Institutes of Health consensus classification system is commonly used to assess prognosis subsequent to surgery (20). However, Yamamoto *et al* (18) found that in KIT-positive EGISTs, the mitotic count, but not the tumor size, was correlated with a worse prognosis. Consequently, the present study adopted their findings to define a grading method on the basis of mitotic count.

Numerous studies have reported the CT and MRI features of primary GISTs, including heterogeneous enhancement, exophytic growth, a size of >5 cm, a necrotic or cystic center, mucosal ulceration, tumor vessels and aneurysmal dilatation (7–10). Metastases are found most commonly in the liver (15.9–34.6%) followed by the mesentery (26%) and peritoneum (11.5–13.0%) (7,9,10). The water-like attenuation or signal intensity in the center of metastases indicates necrosis or cystic degeneration, and the peripheral hypervascular portion represents solid tumor. Lymphadenopathy is not a feature of GISTs (8,9). Calcification, hemorrhage and ascites are rare characteristics in GISTs (7–10). Tateishi *et al* (10) reported that CT findings of a large tumor size ≥11.1 cm, unclear boundaries, an irregular surface, heterogeneous enhancement, the presence of invasion, hepatic metastasis and peritoneal dissemination were favorable for a diagnosis of high-grade GIST and affected the five-year survival rate. Similarly, Ulusan *et al* (7) found that heterogeneous enhancement, size (>10 cm), localization, cystic-necrotic components and metastases were correlated with malignant GIST. To the best of our knowledge, few studies have reported the CT and MRI findings of EGISTs. Due to the rarity of EGISTs, much of the available radiological information is derived from small case series, which identify EGISTs as large masses with solid and cystic components and without an air-fluid level (3–6,11–13,15–19). In the present study, the majority of the EGISTs appeared as round or oval (66.7%; 16/24), cystic-solid (87.5%; 21/24) and ill-defined (66.7%; 16/24) soft-tissue masses. The EGISTs were hypodense (69.6%; 16/23) or isodense (30.4%; 7/23) on CT images, hypointense (50%; 3/6), isointense (33.3%; 2/6) or hyperintense (16.7%; 1/6) on T1WI, and hyperintense on T2WI (100%; 6/6) and DWI (100%; 6/6). These results show that the attenuation and intensity of EGISTs are non-specific and that DWI is unable to differentiate low- and high-grade EGISTs. In the study, 54.2% (13/24) of EGISTs displayed tumor vessels, 95.8% (23/24) of the masses showed marked enhancement and 87.5% (21/24) demonstrated heterogeneous enhancement. Calcification, hemorrhage, ascites and lymphadenopathy were rare signs, and metastases were most common in the liver (37.5%; 9/24). Analyses revealed that tumor size, borders and vessels, and distant metastasis correlate with high-grade EGISTs. The imaging findings of EGISTs in the present study exhibit certain differences compared with those found in GISTs and EGISTs (3,4,7–10). The discrepancy may be caused by the small number of EGISTs or the differing pathological behavior between GISTs and EGISTs.

Differentiation between EGISTs and other intra-abdominal tumors, including benign cystic masses, leiomyosarcoma, malignant fibrous histiocytoma, fibrosarcoma, liposarcoma and solitary fibrous tumors, by radiology is difficult without surgical pathology (2,3). These non-EGISTs may share the majority of imaging characteristics with EGISTs. The present results showed that EGISTs tend to be characterized by certain features, such as advanced patient age, large tumor size, cystic-necrotic components, rare lymphadenopathy, a pattern of heterogeneous enhancement and hepatic metastasis. Also, several radiological characteristics of EGISTs, including the size, borders, tumor vessels and distant metastasis, can provide useful information in the differentiation between low- and high-grade EGISTs.

The present study has several limitations. Firstly, the study is retrospective. Secondly, the number of patients is small. Owing to the rarity of EGISTs, a large multi-institutional study on the radiological diagnosis of EGISTs is therefore required.

In conclusion, EGISTs are rare and aggressive tumors with a predilection for the mesentery, omentum and retroperitoneum. CT and MRI can accurately reveal the location and extent of EGISTs, and certain features, such as advanced patient age, large tumor size, cystic-necrotic components, rare lymphadenopathy, a pattern of heterogeneous enhancement and hepatic metastasis may aid in the diagnosis of EGISTs. Also, radiological characteristics, such as a large tumor size (>10 cm), ill-defined borders, tumor vessels and distant metastasis, can provide useful information in identifying the malignant behavior of EGISTs.

## Figures and Tables

**Table I:** General information of 24 patients with extragastrointestinal stromal tumors.

Variable	Omentum (n=4)	Mesentery (n=19)	Retroperitoneum (n=1)
Gender			
Male	1	11	1
Female	3	8	-
Age, years			
≤40	1	2	-
41–50	2	7	1
51–60	1	4	-
61–70	-	3	-
71–80	-	1	-
≥81	-	2	-
Pathological subtype			
Spindle cell	4	16	1
Epithelioid cell	-	1	-
Mixed cell	-	2	-
Size, cm			
≤5	-	2	-
5–10	-	4	1
>10	4	13	-

**Table II:** Immunohistochemical results of 24 patients with extragastrointestinal stromal tumors.

Result	n (%)
KIT(+)	22 (91.7)
CD34(+)	17 (70.8)
KIT(+) and CD34(+)	15 (62.5)
KIT(+) and CD34(−)	7 (29.2)
KIT(−) and CD34(+)	2 (8.3)

CD, cluster of differentiation.

**Table III:** Computed tomography and magnetic resonance imaging findings of 24 patients with extragastrointestinal stromal tumors.

Criteria	Low-grade (n=7)	High-grade (n=17)	P-value
Localization			
Omentum	1	3	0.678
Mesentery	6	13	
Retroperitoneum	-	1	
Size, cm			
≤5	2	0	0.041
5–10	2	3	
>10	3	14	
Contours			
Round or oval	5	11	1.000
Irregular	2	6	
Borders			
Ill-defined	2	14	0.021
Well-defined	5	3	
Cystic-necrotic component			
Present	6	15	1.000
Absent	1	2	
Hemorrhage			
Present	-	2	1.000
Absent	7	15	
Calcification			
Present	1	-	0.292
Absent	6	17	
Degree of enhancement			
Mild	1	-	0.292
Marked	6	17	
Pattern of enhancement			
Homogeneous	1	2	1.000
Heterogeneous	6	15	
Tumor vessels			
Present	1	12	0.023
Absent	6	5	
Ascites			
Present	1	2	1.000
Absent	6	15	
Lymphadenopathy			
Present	-	1	1.000
Absent	7	16	
Distant metastasis			
Present	-	10	0.019
Absent	7	7	
